# Key populations and Sub-Saharan Africa's HIV response

**DOI:** 10.3389/fpubh.2023.1079990

**Published:** 2023-05-16

**Authors:** Enos Moyo, Perseverance Moyo, Grant Murewanhema, Malizgani Mhango, Itai Chitungo, Tafadzwa Dzinamarira

**Affiliations:** ^1^Department of Public Health, Medical Centre Oshakati, Oshakati, Namibia; ^2^College of Medicine and Health Sciences, University of Zimbabwe, Harare, Zimbabwe; ^3^Department of Public Health, University of Western Cape, Bellville, South Africa; ^4^International Center for AIDS Care and Treatment Program (ICAP) at Columbia University, Harare, Zimbabwe

**Keywords:** key populations, HIV transmission, risk factors, prevention measures, Sub-Saharan Africa

## Introduction

The global HIV epidemic has had the greatest impact on Sub-Saharan Africa (SSA). An estimated 67% of the 38.4 million people living with HIV (PLWH) globally in 2021 were from SSA. SSA was responsible for 670,000 of the 1.5 million new infections and 280,000 of the 650,000 AIDS-related deaths reported globally in 2021. 70% of the new infections reported globally in 2021 were among key populations and their partners. In SSA, key populations accounted for 51% of new infections in 2021 (1).

Key populations (KPs) are population groups who, due to specific higher-risk behaviors and other structural factors, are at an increased risk of contracting HIV, viral hepatitis, or sexually transmitted infections (STIs) (2). They include men who have sex with men (MSM), people who inject drugs (PWID), sex workers, transgender women (TGW), and people in prisons and other closed settings. KPs face several challenges including legal and social issues related to their behaviors that increase their vulnerability to HIV acquisition (2). The risk of HIV infection for a person who injects drugs is ~35 times greater than that of non-injecting adults, 30 times greater for female sex workers than for adult women, 28 times greater for MSM than for other adult men, and 14 times greater for TGW than for other adult women (1). KPs interact with the general population, creating additional chains of HIV transmission and, hence escalating the epidemic. HIV control among KPs is critical for epidemic control. Against this background, we discuss some of the drivers of HIV transmission among KPs in SSA, along with proposed strategies to lower the risk.

## Drivers of HIV infection among key populations

HIV transmission among KPs must be recognized as one of the drivers of the HIV epidemic in SSA. By their nature, no one size fits all solution is available to deal with the challenge. Multipronged, multisectoral interventions are required and are premised upon an understanding of the driving factors that influence HIV infection in the different categories of KPs. While there could be significant overlap, some of the drivers of transmission are unique to specific groups of the KPs.

## Men who have sex with men (MSM)

There are several categories of risk factors for HIV acquisition and transmission in MSM, including those at the individual, biological, community, health system, and structural levels. The number of lifetime male partners, some of whom may be concurrent, low condom use, and high use of injection and non-injection drugs are the individual factors that raise the risk of HIV transmission among MSM (3). Unprotected receptive anal intercourse carries an HIV transmission risk that is almost 18 times greater than penile-vaginal sex (4). The rectum's thin lining, which is prone to laceration during anal sex, as well as the greater density of macrophages that express CCR5 receptors in the rectum compared to the vagina, both contribute to the greater risk of HIV infection among MSM (5).

In SSA, MSM utilize lubricants and condoms at a lesser rate than heterosexual partners. Additionally, there is a greater risk of HIV transmission during anal sex than during vaginal sex due to condom breakage. Many MSM are reluctant to seek STI treatment because they fear being exposed to the public and being rejected by their friends and families (6). In addition, they are reluctant to disclose their sexual orientation due to the stigma and discrimination they encounter from healthcare workers (HCWs), which inhibits them from getting the right STI treatment. HIV transmission risks are increased for both parties when having sex while suffering from an STI. MSM frequently delay seeking HIV care because of the alleged stigma and prejudice from HCWs (7). Because of this, MSM living with HIV delay the initiation of antiretroviral therapy (ART), leading to high viral loads and an increased risk of transmitting HIV (7).

Many SSA nations still consider homosexuality illegal. For fear of being criminalized, MSM restrict their access to HIV prevention and treatment programs (6). As a result of the restrictive legislation, MSM experience violence in their communities such as gang rapes, increasing their chances of contracting HIV (8). They might also hesitate to seek medical attention out of concern of being reported to the authorities. Since they are less likely to receive services like HIV post-exposure prophylaxis (PEP), their risk of contracting HIV is higher (6).

## Sex workers

Some clients of female sex workers (FSWs) in SSA include truck drivers, who have a higher prevalence of HIV than the general population (9). There is also a high prevalence of STIs among FSWs, which increases their risk of contracting HIV (9). Due to the lack of power to negotiate condom use, they end up having unprotected sex, increasing their chances of HIV acquisition. FSWs may also agree to have unprotected sex for fear of violence from their clients, or the fear of losing the clients (10). Some FSWs in the region engage in vaginal cleansing and other practices that keep the vagina dry because they think their customers will have more enjoyable sex as a result (11). The vaginal mucosa is disrupted or inflamed as a result, which raises the risk of HIV transmission. Additionally, some clients may prefer unprotected anal intercourse over vaginal sex, which raises the risk of HIV transmission (11).

FSWs are also more likely to use injectable and non-injectable drugs, which lead to intoxication that may lead to unprotected sex (12). In addition, FSWs may be afraid to seek services at healthcare facilities for fear of stigma and discrimination (6). Even when they are sexually abused, they are unlikely to seek healthcare services for fear of being reported to the police and being jailed, since sex work is illegal in most countries in SSA. The judgemental attitude of HCWs also prevents FSWs from seeking HIV services, resulting in an increased risk of HIV transmission among them (13).

## People who inject drugs (PWID)

There is an association between substance abuse and sexually risky behavior. When people are intoxicated, they are unlikely to use condoms during sex (6). Moreover, many women who inject drugs rely on their sexual partners for access to drugs, so they lack control over the injection equipment (12). Since there are no needle exchange programs in most countries in SSA, PWID usually share used needles, which increases their risk of HIV infection (12). While intoxicated, PWID have a high chance of being sexually abused (6). PWID may also turn to sex work to get money for drugs since they usually have difficulties in keeping other jobs due to drug addiction. Furthermore, as a result of the criminalization of injection drug use in most countries in SSA, PWIDs are unlikely to seek drug abuse rehabilitation services (6).

## Transgender people

HIV infection poses a serious risk to transgender people, particularly TGW. Unprotected sex, sex work, and the use of infected needles for illegal hormone injections have all been related to the elevated risk (14). Transgender people's career alternatives are also restricted due to legal marginalization, which forces them into sex work. While some TGW may need hormonal injections for their physical transformation, due to illegality, most public healthcare facilities in SSA do not offer them. TGW turn to illegal hormone injections as a result, putting themselves at risk of HIV transmission through sharing of contaminated needles. There have also been reports of sexual assault on TGW in the region based on their gender identity (6). Some TGW have low self-esteem as a result of experiences with gender-based discrimination, abuse, victimization, rejection, and social marginalization, which makes it difficult for them to convince their partners to use condoms (7). TGW encounter additional challenges in accessing STI and HIV services which include discriminatory policies, stigma, discrimination by HCWs at healthcare facilities, and criminalization (7).

## People in prisons

The criminalization of injection drug use, sex work, and homosexuality results in the overrepresentation of key populations in prisons in SSA (6). Since these people already have a high prevalence of HIV compared to the general population, they may increase HIV transmission in prisons (6). Most prisons in SSA are overcrowded and experience staff shortages. As a result, there is limited oversight of inmates, resulting in sex, violence, drug use, and tattooing occurring unnoticed (15). Inmates engaging in the abuse of injectable drugs do not usually have an adequate supply of sterile needles and therefore end up sharing them. Inmates share tattooing equipment due to the non-availability of sterilizing equipment, and this puts them at an increased risk of HIV infection. Violence may result in unprotected exposure to blood (15). The demand for basic hygienic items such as soap, toothbrushes, and toothpaste is so high in prisons that some inmates use them to barter unprotected sex. In addition, the sharing of razor blades and toothbrushes is common in prisons in SSA. Condoms and other STI and HIV services are not as easily available in prisons as they are in the general population, and this puts prisoners at an increased risk of HIV transmission (16).

## Strategies to reduce HIV infection among key populations

Strategies that can be used to reduce HIV transmission among KPs include policy reform, socioeconomic empowerment, increasing availability and access to HIV services, and engaging the KPs and their communities (6).

## Policy reform

Countries in SSA should decriminalize sex work, homosexuality, and transgender activities. This will allow KPs not to go underground. The KPs may not experience discrimination at healthcare facilities, which will enable them to seek STIs and HIV services early. Decriminalization will allow KPs to have access to HIV services, which will reduce HIV transmission among them. Early access to HIV care and treatment will result in reduced transmission to their partners once the viral load becomes undetectable (6). Furthermore, decriminalization will potentially reduce violence and sexual abuse faced by these populations since they will be able to report the perpetrators to the police without fear of being arrested. Once there is a policy for decriminalizing the activities of KPs, partner organizations involved in HIV prevention services may reach the populations more easily (17). Although reducing HIV infection among KPs largely depends on policy reform, this remains challenging in most countries in SSA because politicians are afraid of losing the support of religious communities that believe that the activities of key populations are evil. Advocacy is therefore required to change the opinions of key stakeholders for policies to be reformed (17).

## Increasing availability and accessibility of HIV care and treatment services

Since HIV care and treatment services are not easily available and accessible to KPs, governments in SSA should make it a priority to avail the services to these populations. Availability and accessibility can be increased by training of HCWs to ensure that KPs do not experience discrimination (17). HIV services should also be integrated with other medical services such as STI testing and treatment to facilitate access to HIV care and treatment services (18).

## Engaging key populations and communities

Education and engagement of communities about KPs will help ensure that the communities accept key populations. This will reduce discrimination and stigmatization, which will help reduce the sexual abuse and violence perpetrated against the KPs. Acceptance by their communities and families will enable them to get the needed support when taking HIV prevention measures and ART and reduce HIV transmission (10). Furthermore, the engagement of KPs increases their uptake of HIV services (17). Moreover, if these populations are involved from the formulation to implementation of HIV prevention programs, they will take ownership of the programs, resulting in the programs' success (19).

## Empowerment of key populations

Some individuals engage in risky behaviors for survival reasons. To reduce risky behaviors, KPs need financial empowerment to cater to themselves. Discrimination in the workplace must be dealt with. Key populations should also be helped to start income-generating projects. FSWs may benefit from skills training, which will empower them to start their projects and get employment. This will help in reducing sexual abuse and exploitation (19). PWID may benefit from ensuring that drug rehabilitation centers are easily available in the region. Furthermore, needle exchange programs for PWID may ensure that they do not share needles, which is one way in which they get infected with HIV (12).

## Conclusion

Key populations contributed to about half of the HIV new infections in 2021 in SSA. Understanding the risk factors and putting in place strategies to effectively deal with these risk factors are critical elements for the HIV epidemic control in SSA. Therefore, it is important for the different relevant stakeholders involved in HIV control to declare HIV transmission among KPs a global health challenge and expeditiously put in place measures for control. In this article, we provided strategies that can be used to reduce HIV transmission among KPs.

## Author contributions

EM: conceptualization and writing original draft. PM: writing original draft. GM, IC, and MM: writing review and editing. TD: conceptualization, supervision, and writing review and editing. All authors contributed to the article and approved the submitted version.
